# Bacteriocin diversity, function, discovery and application as antimicrobials

**DOI:** 10.1038/s41579-024-01045-x

**Published:** 2024-05-10

**Authors:** Ivan Sugrue, R. Paul Ross, Colin Hill

**Affiliations:** 1 APC Microbiome Ireland, University College Cork, Cork, Ireland; 2 School of Microbiology, University College Cork, Cork, Ireland

## Abstract

Bacteriocins are potent antimicrobial peptides that are produced by bacteria. Since their discovery almost a century ago, diverse peptides have been discovered and described, and some are currently used as commercial food preservatives. Many bacteriocins exhibit extensively post-translationally modified structures encoded on complex gene clusters, whereas others have simple linear structures. The molecular structures, mechanisms of action and resistance have been determined for a number of bacteriocins, but most remain incompletely characterized. These gene-encoded peptides are amenable to bioengineering strategies and heterologous expression, enabling metagenomic mining and modification of novel antimicrobials. The ongoing global antimicrobial resistance crisis demands that novel therapeutics be developed to combat infectious pathogens. New compounds that are target-specific and compatible with the resident microbiota would be valuable alternatives to current antimicrobials. As bacteriocins can be broad or narrow spectrum in nature, they are promising tools for this purpose. However, few bacteriocins have gone beyond preclinical trials and none is currently used therapeutically in humans. In this Review, we explore the broad diversity in bacteriocin structure and function, describe identification and optimization methods and discuss the reasons behind the lack of translation beyond the laboratory of these potentially valuable antimicrobials.

## Introduction

Antimicrobial peptides are produced ubiquitously across the kingdoms of life as an ancient form of host defence^1^. Bacteriocins are a subset of bacterially produced, ribosomally synthesized antimicrobial peptides that have been studied for almost a century^2^. In that time, bacteriocins have been the subjects of thousands of publications, describing diverse peptide families, many of which have reported the ability of the peptides and/or their producers to control the outgrowth of food spoilage organisms and to preserve foods^3^. Many bacteriocins are the products of safe and food grade fermenting species such as lactic acid bacteria, and some, exemplified by nisin, have transitioned in the past century from unknown antimicrobial by-products^2^ to characterized^4^, regulated and approved natural food additives^5^ in widely consumed commercial products. Bacteriocins have found success as food preservatives in the present but are not limited to it for the future, as they have also been investigated for various applications such as growth promotion in production animals^6^, plant growth promotion^7^ and clinical therapeutics for the treatment of fungal^8^, protozoal^9^, viral^10^ and, foremostly, bacterial infections^11^.

The increasing rates of antimicrobial resistance threatens the use of current antibiotics, and it is predicted that antibiotic-resistant microorganisms will cause millions of deaths worldwide every year by 2050, severely affecting what are now considered routine medical treatments^12^.

The WHO has generated a priority pathogen list highlighting an urgent need for novel treatments against antibiotic-resistant pathogens, with special mention of *Mycobacterium tuberculosis* and carbapenem-resistant Gram-negative bacteria for which only a few treatment options are available, followed by the Gram-positive vancomycin-resistant *Enterococcus faecium* and other high-priority pathogens^13^. Bacteriocins represent a potential solution as current antibiotic alternatives or adjuncts^14–17^. They offer several pharmacological advantages such as activity against pathogens in the nanomolar and picomolar ranges, narrow spectra, general non-toxicity and distinct mechanisms of action^18^. The diversity in bacteriocin structures and mechanisms also represents a range of ribosomally produced antimicrobials that are amenable to genetic engineering strategies enabling the production of bioengineered variants with superior efficacy against target microorganisms^19^.

As natural agents of microbial competition, bacteriocins offer an advantage to bacteria over sensitive neighbouring species, enabling invasion, colonization and protection of a niche in complex environments such as the gut microbiome^20^. By exploiting these natural mechanisms, they can be used as a tool for humans to deliver antimicrobial activity as pure peptides and composite nanoparticles or can be codified within live biotherapeutics for in situ expression in microbiomes. Advances in microbiome metagenomics and other ‘omics’ technologies over the past two decades have revealed catalogues of genome-mined and metabolome-mined bacteriocins, and new families are continually being discovered, but currently discovery is outpacing characterization and application. Narrow-target spectrum bacteriocins are also increasingly desirable as we advance our understanding of host– microbiota interactions (response and regulation of host processes to microbiota composition and products^21^) and are realizing that the impact of antibiotics on microbiome diversity should be considered^22^. Despite lack of investment, bacteriocins are currently undergoing clinical studies to treat infections caused by antimicrobial-resistant pathogens; the outlook is bright for diverse bacteriocin applications in the next century.

In this Review, we summarize recent advances in bacteriocin research, including the identification of novel peptides and mechanisms of action. We discuss bioengineering as a means to optimize bacteriocin activity and expression in addition to novel methods of bacteriocin discovery in the omics era. Finally, we outline the potential applications of bacteriocins producers, including live biotherapeutics for microbiome modulation, and highlight those peptides that are currently under investigation for clinical applications.

## Bacteriocin diversity

### Structural diversity

**Fig. 1:**
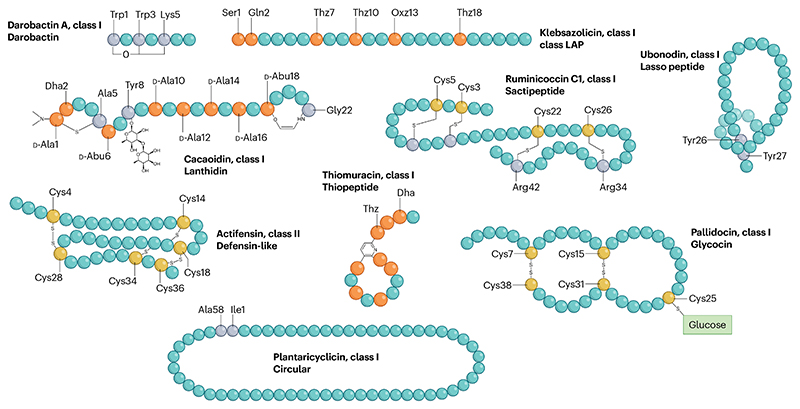
The structural diversity of bacteriocins. Bacteriocins are typically classified into classes and subgroups based on their mechanism of production and secondary structures (Table 1). Lanthipeptides and other class I bacteriocins may contain complex intrapeptide bonds that are installed through enzymatic modifications of specific residues in the peptide backbone. These bonds may form small ring structures such as the C–O–C Trp–Trp ether bond in darobactin A (key residues coloured in grey) and the cacaoidin AviMeCys ring (modified residues coloured in orange). Multiple intrapeptide crosslinks may be formed following modification within one peptide such as the sacthioine bonds in ruminicoccin C1 (involved cysteine residues coloured in yellow). Class I bacteriocins may also feature post-translational installation of other moieties, shown here in glycosylated pallidocin at cysteine 25 and tyrosine 8 of cacaoidin. Modified residues are not always part of larger ring structures, such as the thiazole and oxazole residues present in the linear azoline-containing peptide, klebsazolicin. Large ring structures, however, are prominent features of different subgroups of class I bacteriocins and take different forms such as the loop structure present in the lasso peptide ubonodin that is sterically locked by bulky tyrosine side chains or the macrocycle of the heavily modified thiomuracin A. The largest rings are present within the circular bacteriocins wherein N-terminal and C-terminal residues are covalently linked resulting in a closed loop structure. Class II bacteriocins do not undergo enzymatic modification and are therefore less enzymatically decorated but may feature disulfide bonds that can also result in intercalated structures of such as those in defensin-like bacteriocins, such as actifensin. LAP, linear azole-containing or azoline-containing peptide.

Bacteriocins are diverse and widespread across the phyla that produce them, with enormous heterogeneity in gene cluster organization, bio-synthetic machinery and peptide structures (Table 1 and Fig. 1). Despite such diversity, bacteriocins are usually classified into two major groups: those with post-translational modifications (class I) and unmodified peptides (class II) (Box 1). In recent years, there have been increased reports of novel ribosomally produced and post-translationally modified peptides (RiPPs) and characterization of their biosynthetic machinery (extensively reviewed elsewhere^23^). However, not all RiPPs are bacteriocins and nor are all bacteriocins RiPPs as some do not undergo substantial post-translational modification. Bacteriocins are generally considered large molecule antimicrobials relative to small-molecule antibiotics but can range from bicyclic peptides, such as the recently described darobactin (965 Da)^16^ that is smaller than the clinically used colistin (~1,200 Da)^24^, to larger circular peptides such as pumilarin (7,083 Da)^25^ (Fig. 1).

Class I bacteriocins undergo extensive modification with enzymes that may dehydrate residues and form inter-amino acid bonds, leading to their intricate and stable structures. Such modifications can result in peptides that are extensively cyclized and dehydrated, such as polyheterocyclic klebsazolicin^26^ and corynaridin, the latter of which contains eight dehydrated residues^27^. Lantibiotics (lanthionine-containing antibiotics) are well-established class I bacteriocins, but novel structurally related peptide families continue to be identified; such as the type V lanthipeptides (lanthidins), which are glycosylated and N-terminally dimethylated, as evident in the founding member cacaoidin^28^. Class II bacteriocin structures include simple linear α-helical peptides such as plantaricin EF^29^, peptides with conserved complex secondary structures such as the four-helix peptide BacSp222 (ref. 30) or linear peptides with specific conserved disulfides such as pediocin-like maltaricin CPN and actifensin^31,32^. Class II bacteriocins do not feature the substantial modifications, such as dehydration and formation of unusual amino acids or linkages of class I, and previously included circular bacteriocins that are subject to N-terminal to C-terminal covalent linkage resulting in their simple circular shape^33^ and the microcins that are post-translationally conjugated to the siderophore (iron binding) moiety^34^. For consistency within the two major classification schemes (Box 1), we suggest that circular and siderophore-modified peptides are henceforth referred to as class I bacteriocins, as they undergo modification despite relatively simple mature structures.

For most bacteriocins, the structural peptide-encoding gene also encodes a leader sequence, which comprises N-terminal residues removed from the mature peptide, and can fulfil various functions in peptide export, cleavage and modification^23^. These roles are linked to the specific type of bacteriocin they accompany, shaping peptide production and post-translational alterations. For example, in the case of lantibiotics, conserved motifs such as the FNLD motif are observed within leader sequences, whereas circular bacteriocins exhibit considerable diversity in their leader sequences, ranging from minimal lengths as seen in lactocyclin Q (two residues^35^) to more extensive sequences such as in amylocyclicin (47 residues^36^). The flexibility of circular bacteriocin leader sequences was highlighted by modification of circularin A, which demonstrated functional expression with a leader of only one residue (Met), suggesting a limited role for the leader in active circularin A biosynthesis^37^. Additionally, specific motifs within the nisin leader sequence have been shown to guide peptide export, modification^38^ and cleavage^39^, whereas in lanthipeptides, such as mersacidin, the leader undergoes a two-step cleavage process involving initial cleavage upon exiting the cell by the transporter-peptidase MrsT^40^ and subsequent cleavage outside the cell by specific extracellular proteases such as AprE^41^. Although many bacteriocins with leader sequences possess a dedicated leader sequence essential for their biosynthesis and activity, with exceptions such as circularin A described earlier, leaderless bacteriocins lack this and instead are directly translated as active peptides^33^ and the mechanisms of their production and export are poorly understood. Two pleckstrin homology domain-containing proteins DdE and DdF have been linked to the export of enterocin DD14 peptides in *Enterococcus faecalis*^42^, but peptide export mechanisms are yet to be characterized.

### Biosynthetic gene clusters

Bacteriocin-encoding gene clusters encode essential factors involved in their production and protection, typically incorporating genes encoding a core peptide, modifying enzymes for modified peptides^43^, export machinery, self-immunity proteins that protect the producer^44^ and many clusters also contain genes encoding regulatory proteins^45^. In the case of the unmodified bacteriocin bactofencin, a ~4 kb gene cluster encodes the peptide and an ABC transporter with a protease domain that exports and removes the leader sequence. These two genes alone (*bfnA* and *DLSL_0052*) have been demonstrated to produce active peptide when expressed heterologously in *Escherichia coli*^46^. By contrast, the native producer of bactofencin A, *Ligilactobacillus salivarius* DPC6502, produces an immunity protein that provides self-protection and an additional protein containing a thioredoxin family domain that is hypothesized to assist in disulfide bond formation^46^. Other small gene clusters result in more complicated peptide structures such as a cluster of 6.2 kb that contains genes for darobactin A production: a core peptide (*darA*), an ABC transporter (*darBCD*) and a single-radical *S*-adenosylmethionine enzyme (encoded by *darE*) that catalyse the formation of a Trp–Lys C–C bond and a C–O–C Trp–Trp ether bond via an unknown mechanism^16^.

Radical *S*-adenosylmethionine enzymes also catalyse the formation of α-carbon to thioether (sactionine) bonds in sactibiotics (sactionine-containing antibiotics), such as the ruminococcins, in which the gene cluster in *Ruminococcus gnavus* E1 consists of 17 open reading frames over 15 kb that can result in five distinct peptides^47^. In the case of the lanthidin cacaoidin, the gene cluster spans 30 kb and contains 27 genes that encode the necessary enzymes for multiple modifications, including rhamnose synthesis and glycosyl transferases, dehydration and C-terminal *S*-[(Z)-2-aminovinyl-3-methyl]-d-cysteine (AviMeCys) formation^28^. Many bacteriocin gene clusters are not constrained to specific phyla, such as glycocins and lasso peptides, which are encoded within the genomes of both Gram-positive and Gram-negative bacteria^48,49^. Bacteriocin gene clusters may be present on plasmids capable of conjugation or transposable elements, thus facilitating transfer of the bacteriocin production phenotype in a competitive environment^50^. Recently, it was found that up to 3% of prophage encode biosynthetic gene clusters, the majority of which are for bacteriocin production and can demonstrably transfer production phenotype to nascent strains^51^.

### Functional diversity

Although this Review mainly focuses on the antibacterial nature of bacteriocins, they are known to have a diverse set of other biological functions. A wide range of peptides possess antiviral activities^52^ such as subtilosin from *Bacillus amyloliquefaciens* that displays antiviral activity against herpes simplex viruses^53^. Peptides have been demonstrated to have cytotoxic effects towards cancer cell lines such as Laterosporulin10 (ref. 54) or possess in vivo antitumour activity as was shown for microcin E492 against human colorectal cancer cells using a xenografted zebrafish model^55^. Bacteriocins can modulate immune systems such as sublancin that can enhance phagocytic activity of macrophages and increase T cell frequency in mesenteric lymph nodes in mouse models of methicillin-resistant *Staphylococcus aureus* infection^56,57^. Thuricin-17, which is produced by *Bacillus thuringiensis*, has been shown to promote the growth of canola plants (*Brassica napus* L.) at high temperatures through an unknown mechanism, displaying a potential role for bacteriocins in supporting plant growth in changing climates^7^. Finally, human streptococcal pathogens co-regulate complex bacteriocin production and competence pathways linking fratricide and natural transformation, implicating a role for bacteriocins in gene acquisition^58^.

## Narrow-to-broad spectrum peptides

**Fig. 2:**
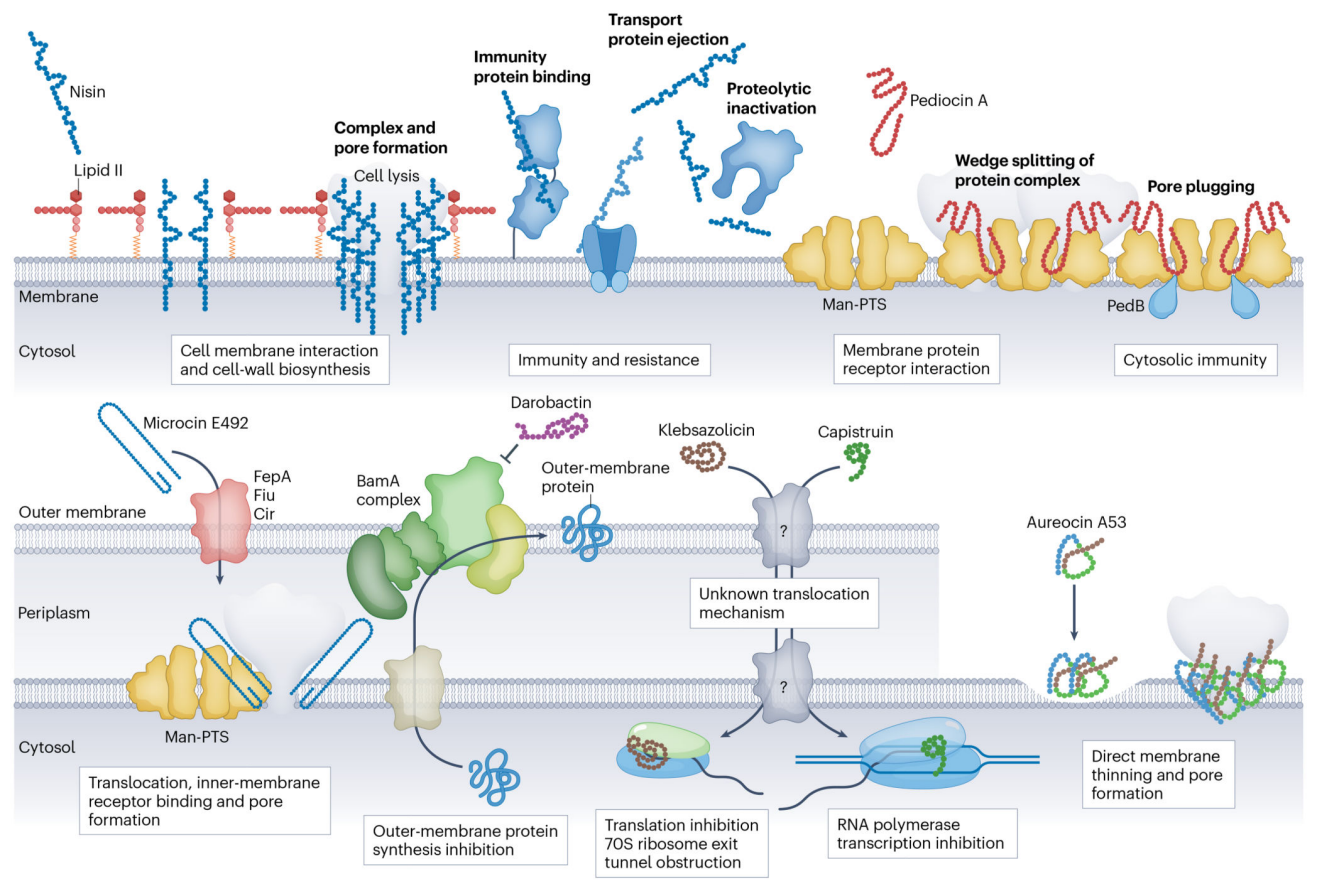
Bacteriocins mechanisms of action and resistance mechanisms. Bacteriocins are heterogeneous in function and exhibit various mechanisms of action. Bacteriocins are typically cationic hydrophobic peptides that are drawn towards anionic bacterial cell surfaces. At the surface, they can interact with specific receptors, such as nisin that complexes specifically to the cell-wall precursor molecule lipid II and subsequently forms pores across the membrane, which leads to cell death. Mechanisms of immunity and resistance protect strains from nisin by binding, ejecting or proteolytically cleaving the peptides. Pediocin-like bacteriocins (class II) attach to the extracellular surface of the mannose-phosphotransferase system (Man-PTS) core domain, penetrate the membrane and shift the core domain away from its V-motif, thereby forming a pore in the membrane. PedB proteins in the targeted bacterium can physically plug the pore formed by pediocin PA-1 and the Man-PTS, thereby providing cytosolic immunity. Other peptides target the membrane directly, such as aureocin 53 that initially causes Gram-positive membrane thinning by accumulating in the upper leaflet of the phospholipid bilayer, which is mediated by specific α-helices of the peptide, and then causes poration favoured by other α-helices within the peptide. Bacteriocins can also interrupt essential cellular processes such as outer-membrane protein integration of BamA by darobactin or inhibition of protein and nucleic acid synthesis of klebsazolicin and capistruin, respectively. Microcin E492 translocates across the outer membrane using a ‘Trojan horse’ strategy, wherein the siderophore moiety is recognized by outer-membrane transporters that facilitate uptake of the peptide into the periplasm, followed by binding to the mannose-PTS at the inner membrane and subsequent lethal disruption of membrane potential. Immunity to microcin E492 is provided by the action of the immunity protein MceB, via an unknown mechanism.

Bacteriocins, such as antimicrobial peptides across all kingdoms of life, are typically cationic hydrophobic peptides, many of which are active at negatively charged bacterial cell surfaces^59–61^. The activities of bacteriocins, once they reach the cell surface, are diverse, including specific interactions with cell-wall or cell-membrane components, general membrane interaction and pore formation, or receptor-mediated cell penetration and interference with intracellular processes (for extensive mechanistic reviews, see refs. 62–64) (Fig. 2).

### Extracellular targets

Nisin is well established to have a dual mechanism of action, binding the cell-wall precursor molecule lipid II, thereby inhibiting peptidoglycan biosynthesis and subsequent pore formation leading to cell death^65^. Initially, it was believed to form a pore complex of eight nisin and four lipid II molecules, although more recent evidence has shown that the pore complexes continue to grow causing massive cell membrane damage^66^ (Fig. 2). Numerous post-translationally modified bacteriocins share a conserved nisin-like N-terminal lipid II binding domain (rings A and B), but do not possess the same C-terminal domain, and do not form pores as a result^67^. This has been recently been demonstrated for the nisin-like peptides rombocin A^68^ and cesin^69^. Many bacteriocins do not target lipid II and instead use a different receptor or lipid component as a binding site^70,71^.

Box 1 **History of bacteriocin nomenclature**

Searching the literature with the term bacteriocin returns more than 11,000 publications, describing a heterogenous group of compounds that mostly share two general features: bacterial ribosomally synthesized peptides/proteins that are antagonistic towards other bacteria. These compounds can range from multiprotein contractile nanomachines^191^ to antimicrobial secreted peptides^192^. The lack of a precise definition after a century of bacteriocin research results in a field that can often be contradictory and confusing.

Jacob et al.^193^ first coined ‘proteins of the colicin type’ as bacteriocins following the initial work of Gratia^194^ in 1925 describing colicins as the source of strain-specific antagonistic activity between *Escherichia coli* (see the figure). Subsequent publications used bacteriocin to describe lytic proteins from Gram-positive bacteria, such as megacin from *Bacillus megaterium*^195^. The discovery of nisin was published in the same decade as colicin^2^ but Gram-positive bacteria-derived antimicrobials were not as widely described as bacteriocins for decades^196^. In 1976, Tagg et al.^196^ acknowledged that no universally accepted definition existed and criticized the use of the term bacteriocin for poorly characterized compounds. It was proposed that bacteriocin should describe bactericidal compounds containing a biologically active protein moiety. The 1993 Klaenhammer scheme expanded this definition, which is the basis for modern bacteriocin classification. Peptides were grouped into class I (post-translationally modified) and class II (unmodified), with larger proteins grouped as class III (ref. 197). Research on bacteriocins from Gram-positive bacteria grew considerably, and in 2005 Cotter et al.^198^ suggested that bacteriocin be reserved for peptides rather than heat-labile proteins. The term has not ceased to be used for these larger proteins. Other schemes have been proposed including up to four classes, and many bacteriocins are now popularly referred to as RiPPs (ribosomally produced and post-translationally modified peptides)^3,23,199^. We suggest consistent usage of the two major class schemes for bacteriocins to provide clarity for future researchers navigating the literature.

**Figure:**
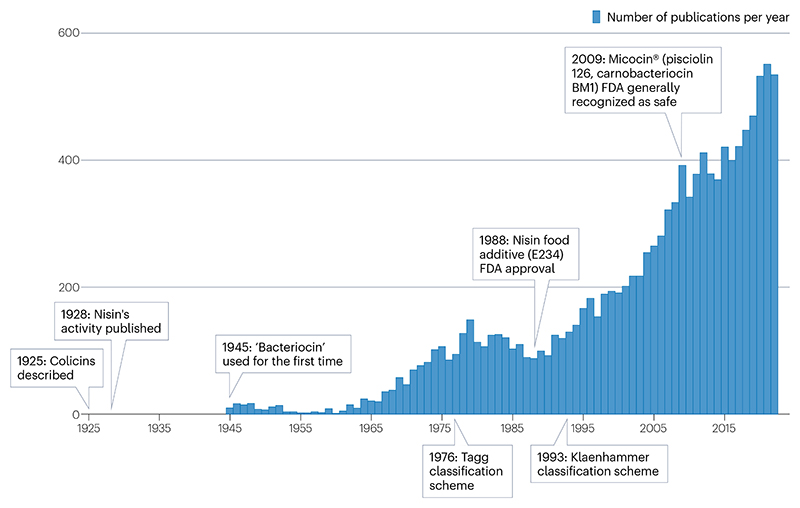

Pediocin-like bacteriocins (class II) interact with the mannose phosphotransferase system (Man-PTS) forcing the channel into an open state, which was recently confirmed by cryo-electron microscopy^72^. The Man-PTS transporter is a receptor for several class II bacteriocins, such as lactococcins A and B and garvicins A, B and C, as was recently confirmed by deletion mutants, protein modelling and cryo-electron microscopy^73,74^, and is the target for microcin E492 after ‘Trojan horse’ translocation across the outer membrane^75^. The circular bacteriocin garvicin ML relies on the maltose ABC transporter to target *Lactococcus lactis*, whereas AS-48 is believed to interact based solely on electrostatic and hydrophobic interactions to insert directly into the lipid bilayer, leading to destabilization^76,77^. Direct membrane interaction is also the mechanism for the four helix-bundle bacteriocins epidermicin NI01 and aureocin A53, which have been found to have distinct multimodal ‘flowering’ pore-forming activity^61^.

Bacteriocin activities are typically restricted within the Gram status of the producer; that is, peptides derived from Gram-positive bacteria are typically far less active against Gram-negative bacteria without assistance of an outer-membrane permeabilizing agent^78,79^. Contradictory to this, a semipurified supernatant containing bacteriocins from *Lacticaseibacillus paracasei* CNCM I-5369 was found to be active against a panel of Gram-negative bacteria, which was confirmed by heterologous expression^80^. Bioengineering and rational design have been utilized in the absence of an outer-membrane permeabilizing compound (which would typically be required for Gram-positive-derived bacteriocins to inhibit Gram-negative bacteria) to produce variants of nisin with up to 12-fold greater activity including bactericidal activity against *E. coli* and *Acinetobacter baumanii*^81^. A hybrid peptide consisting of enterocin CRL35 fused with microcin V (Ent35–MccV) was shown to be active against the targets of both individual peptides, including *Listeria monocytogenes* and *E. coli*^82^.

### Intracellular targets

Some bacteriocins interfere with essential intracellular processes, in particular those that target Gram-negative bacteria, which lack exposed peptidoglycan and inner cell membrane components. The TOMM klebsazolicin inhibits protein synthesis by blocking the ribosome exit tunnel, but its extracellular uptake mechanism is unknown^26^. The crystal structures of the lasso peptides microcin J25 and capistruin have revealed their specific sites of transcription inhibition within the RNA polymerase secondary channel following uptake mediated by a host of transport proteins^83^. Sactibiotics are a class known for their narrow spectra of inhibition. Recently, it was suggested that the *R. gnavus-* produced and *Clostridioides-* targeting sactibiotic ruminicoccin C could act through nucleic acid synthesis inhibition in a manner similar to metronidazole, but this has yet to be confirmed^47^.

### Unknown targets

Despite ongoing characterization of the mechanisms of action of bacteriocins, many peptides of interest remain uncharacterized. Similarly, the recently described bacteroidetocins, a family of anti-Bacteroidales pediocin-like bacteriocins, have an unknown mechanism of action as *Bacteroides* species lack the mannose-PTS^84^. Although most research to date has focused on mechanisms of bacteriocins as free agents in liquid culture, a two-component Gram-negative bacteria-derived bacteriocin CdzCD was recently identified on the basis of cell-to-cell contact-dependent killing, for which the outer-membrane protein PerA was required for sensitivity, and has been suggested as the target receptor for CdzCD binding^85^. With vast recent improvements in protein structural modelling and prediction, we may begin to see more in-depth bacteriocin–target complex structures and gain insights into yet unknown mechanisms.

### Immunity and resistance

The risk of target strains developing resistance against bacteriocins because of continuous use is a key concern, given the current situation with antimicrobial resistance and the antimicrobial arms race. Bacteriocin producers are self-protected by the action of immunity proteins, which may bind or export the peptides^44^, or block the action of peptides on protein complexes^72^. Resistance to bacteriocin activity can occur through general mechanisms, such as cell-wall thickening causing resistance to the lipid II binding lasso peptide, siamycin I^59^, or by truncation of a protein target similar to the transporter of the mannose-PTS^86^ but high-level resistance occurs through acquisition of specific resistance genes. Bacteriocins can be inactivated owing to the activity of specific enzymes such as the nisin resistance protein, which degrades nisin^87^. Worryingly, some pathogenic species express generalized transporter proteins such as *Streptococcus agalactiae* bacitracin type efflux protein (SaNsrFP) that protects against numerous cell-wall targeting compounds including lantibiotics^88^. As bacteriocins have not been extensively used in the clinical setting, the extent or risk for resistance in clinical use is unknown and to our knowledge for many classes of bacteriocins, no resistance mechanisms have been characterized yet.

## Production and bioengineering

**Fig. 3:**
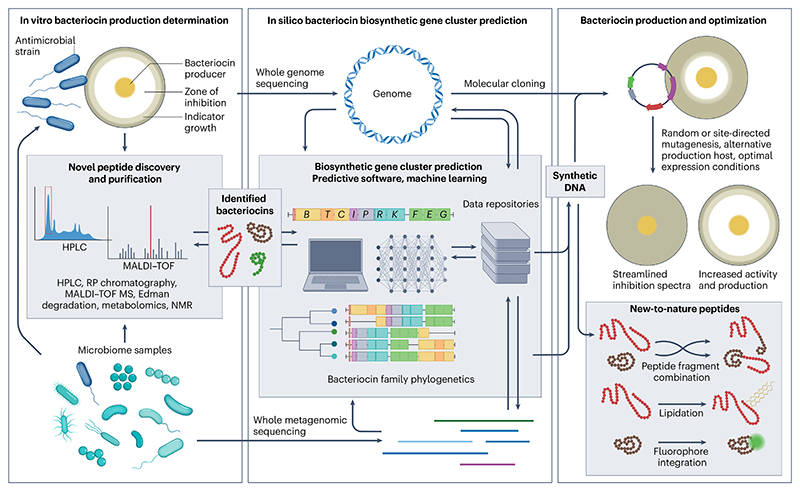
Methods of in silico bacteriocin identification and translation to in vitro production, optimization and bioengineering. Until the recent proliferation of large-scale whole metagenomics, bacteriocins were mainly identified in vitro through screening with agar-based assays. Specific antimicrobial peptides could be identified and purified through a number of techniques including mass spectrometry and HPLC (left panel) and corresponded to a biosynthetic gene cluster identified from a sequenced genome (middle panel, top). Now large data sets can be more easily generated or sourced from data repositories and mined for bacteriocin gene clusters with improved predictive software and corresponded with metabolomic data to identify peptides (left panel, bottom and middle panel). In silico mined peptides and known peptides can be heterologously expressed in desirable hosts to optimize production and/or modified to generate mutant peptides with desirable characteristics such as improved activity or streamlined inhibition spectra (right panel, top). Novel techniques have also been developed to further modify expressed peptides through recombination of peptide fragments generating new-to-nature peptides or modification with compounds of specific properties, such as lipids or fluorophores (right panel, bottom). HPLC, high-performance liquid chromatography; MALDI–TOF MS, matrix-assisted laser desorption ionization–time-of-flight mass spectrometry; RP, reverse phase.

One of the most appealing attributes of bacteriocins is their gene-encoded nature, which enables the alteration of peptides using simple techniques and the generation of new-to-nature structures with unknown characteristics (Fig. 3). Wild-type bacteriocin producers often generate low bacteriocin yields because production is a high-energy process for the cell and is thus tightly controlled. This can result in excessive production costs that render bacteriocins non-viable commercially^89^. Production optimization has therefore been examined by altering growth parameters in addition to exploiting their gene-encoded nature by manipulating the native producers^90^ and/or peptide expression in more efficient or peptide-insensitive heterologous hosts^91,92^.

### Heterologous expression optimization

Bacteriocins of all classes have been subject to heterologous expression to express novel silent genes^93^, understand their mechanism of biosynthesis^94,95^ or to improve their production for commercial viability^92^. Although post-translationally modified (class I) bacteriocins require essential modification enzymes to produce active mature peptides, class II bacteriocins are particularly suited to heterologous expression as many are ‘simple’ linear peptides. A range of Gram-positive bacteria, Gram-negative bacteria, eukaryotic hosts and diverse plasmids have been exploited for this such as *E. coli* for expression of pediocin PA-1 (syn. pediocin AcH), bactofencin A and lactocin AB^46,96^, *L. lactis* for expression of plantaricin JK and enterocin NKR-5-3B^97,98^ and the yeasts *Pichia pastoris* (syn. *Komagataella phaffii*) and *Kluyveromyces lactis* for the expression of enterocins^99,100^.

Beyond heterologous expression, simple optimization of culture conditions can increase bacteriocin yield, as demonstrated by researchers by improving garvicin KS production from *Lactococcus garvieae* as high as 60-fold when cultured in pasteurized milk and tryptone, compared with GM17 media^90^. Combining the improved media with increased gene dosage of the core peptide of the native producer further increased production to the high concentration of 1.2 g of peptide per litre^90^. Increasing gene copy number has also improved production of the sactibiotic thurincin H, in which an additional three copies of the structural gene, *thnA*, resulted in double the yield from broth culture^101^. Synthesis of plantaricin E/F in *Lactobacillus plantarum* strains is induced by both an auto-inducing peptide PlnA1 and acetate via a distinct interaction with the PlnB1 response regulator^102^. Exploiting this mechanistic understanding enabled an increase in the yield of plantaricin in culture by addition of sodium acetate at a concentration of 1 g l^−1^ (ref. 102). Limited studies report extensive optimization or comparative purification yields, as peptide purification is typically a means of characterizing peptides at small scale in vitro rather than for potential commercial application. However, to scale up to in vivo experiments with pure peptide and for bacteriocin production to become more commercially viable, optimization must be explored in greater detail.

### Expanding the functional diversity of bacteriocins

Nisin is perhaps the most genetically manipulated bacteriocin with thousands of unique variants having been generated using random and site-direct PCR mutagenesis techniques, in addition to an entire expression system developed based on the tight regulation of nisin production^103^. Mutagenesis methods have produced nisin variants with increased activity against biofilms and variants with greater activity against Gram-negative bacteria such as *Cronobacter sakazakii, Salmonella typhimurium* and *E. coli*^104^. Rombocin K, a bioengineered variant of nisin-like rombocin A, was found to have a twofold lower minimum inhibitory concentration than the original peptide when tested against *Listeria monocytogenes*^68^. The inhibition spectrum of nisin has also been refined in the derivatives M17Q, T2L and HTK, which possess increased anti-*Staphylococcus* activity and reduced anti-*Lactococcus* activity^19,105^. Similarly, saturation mutagenesis of mutacin 1140 generated 418 single amino acid variants of which Phe1Ile ultimately emerged as the most promising in vivo candidate for *Clostridioides difficile* infection and associated diarrhoea, which in turn underwent a second round of mutagenesis optimization resulting in a more potent peptide^106,107^. Such methods may improve a promising bacteriocin for application or be used to overcome a limitation, such as the oxidation-resistant M31L variant of pediocin PA-1, in which oxidation-associated activity reduction challenges the wider application of wild-type peptide^108^. Bioengineering can be used as a means to overcome proteolytic degradation, such as nisin S29P that is immune to the action of the nisin resistance serine protease^109^ and cesin R15G that has improved resistance to trypsin^69^.

Recently developed methods have utilized incorporation of non-canonical amino acids into the peptide backbone, which can then be conjugated to other compounds via ‘click chemistry’. Using the non-canonical and more-hydrophobic amino acid ethionine in the place of methionine, heterodimeric and fluorescently labelled nisin variants have been generated, each of which retained antimicrobial activity^110^. A similar method lipidated nisin variants, which had improved bio-activity and specificity against pathogenic strains^111^. Conjugation of bacteriocin peptides to molecules such as fatty acids can be exploited to produce bacteriocins that form colloidal stable assemblies, a feature which could be utilized to overcome problems with solubility of hydrophobic peptides^112^. Novel methods such as this can be used to further expand, edit and hone already diverse peptide functionality and future downstream applications.

## Discovery in the meta-omics era

In recent years, bacteriocin discovery has been catapulted into the meta-omics era with the availability of vast genomic data sets and dedicated bioinformatic software enabling in silico screening of bacterial genomes and large-scale metagenomic mining for the presence of novel bacteriocin gene clusters (Fig. 3). Despite convincing evidence that bacteriocin-encoding genes are widely distributed across many genera, confirming bacteriocin production from individual strains isolated during screening studies has proven difficult. Before the increased accessibility of next-generation sequencing technologies, the majority of novel bacteriocins were identified in vitro through laborious screening of hundreds or thousands of bacterial isolates^113^. These culture-dependent screening methods face logistical microbiological limitations such as the choice of media on which samples are plated, the choice of indicator species and the growth conditions (pH, atmosphere composition and temperature), which can all lower the likelihood of bacteriocin detection. Failure to detect bacteriocin production does not mean lack of bacteriocin presence, but could be due to the inadvertent choice of an insensitive indicator species or a limited or lack of induction and production of an active bacteriocin. Genomic approaches that are agnostic of active expression and rely on bioinformatic predictions of the presence of biosynthetic gene clusters can overcome these issues, but are not without their own limitations. For example, in silico approaches alone do not confirm biological activity nor do they enable the purification and characterization of a bacteriocin. It is likely that a combination of in silico and culture-dependent methods will prove most effective in terms of identifying and isolating novel functional bacteriocins.

### Bacteriocin gene cluster prediction

As sequencing technology has improved and sequence databases have grown, a range of tools have been developed for mining bacteriocin genes encoded in bacterial DNA. Tools have been developed that span from ‘point-and-click’ interfaces suitable for non-bioinformaticians to command-line controlled packages to detect biosynthetic gene clusters in genomic and metagenomic data sets at a range of assembly levels. BAGEL and antiSMASH are well-tested and frequently updated tools that are designed for the identification of bacteriocin (and secondary metabolite) biosynthetic gene clusters in assembled bacterial genomes^114,115^. Both tools are useful for the characterization of an unknown antimicrobial-producing isolate but are limited by predictions based on enzyme homology and predicted core peptide characteristics, which may miss truly novel compounds. In addition, they can be low to medium throughput based on the skill level of the user. Despite this, they have enabled the discovery of bacteriocins in several hundreds to thousands of genomes, such as the identification of 101 lanthipeptide gene clusters from 223 *Bacillus cereus* strains^116^, the characterization of the biotechnological potential of 213 diverse lactic acid bacteria^117^ and the genome-guided expansion of a bacteriocin family from an initial 11 members to widespread detection of homologues in hundreds of strains^25^. More recently, a simple tool for mining specific RiPP classes based on BAGEL has been developed to interrogate metagenomic data sets for bacteriocin biosynthetic gene clusters^118^.

With continuous growth in computing power and bioinformatic tool development, artificial intelligence has also been introduced into bacteriocin research, with machine learning being utilized to predict biosynthetic gene clusters and predict chemical structures following post-translational modification. RODEO (Rapid ORF Description and Evaluation Online) was the first tool available to use machine learning and was able to expand the number of known lasso peptides 10-fold upon its initial publication and subsequently increased the number of known glycocins from 6 to 10 distinct characterized members using only a single enzyme as a starting point^49,119^.

### From in silico to in vitro

Bioinformatics can have an important role in identifying potential bacteriocins, but this should be supported by functional characterization by isolation of a producing strain, chemical synthesis or heterologous expression from genomic or synthetic DNA in a suitable host. When candidate bacteriocin-producing strains are identified in silico but production cannot be demonstrated, or when putative producers are not available, *E. coli* has been used as a heterologous bacteriocin expression ‘workhorse’. Expression in *E. coli* strains has enabled the characterization of structural peptides and biosynthetic machinery for glycocins and lantibiotics^40,120,121^. Bioinformatically mined class II bacteriocins have also been expressed in *E. coli*, including pediocin PA-1 and lactobacilli*-* derived pediocin-like peptides, enabling the identification of genes essential for bacteriocin production^46,93^. Gram-positive hosts have also been extensively utilized for the expression of bacteriocins, including the reliable nisin-inducible expression system in *L. lactis*, which has recently been used for the functional characterization of the circular bacteriocin circularin A mined from a clostridial genome^95^. Genome mining paired with heterologous expression is a powerful combination to explore novel compounds but can be low throughput and failure-prone depending on the choice of the host and vector. Researchers recently developed a powerful platform combining high-throughput bioinformatics and an integrated robotic system to express 96 RiPP gene clusters from diverse bacterial phyla involving 383 bio-synthetic genes, with a success rate of 86% (ref. 122). High-throughput methods such as this could be used to explore the Integrated Microbial Genome Atlas of biosynthetic Gene Clusters (IMG-ABC) database that consists of 20,000 gene clusters, of which a small number has been experimentally validated^123^.

### Integrating omics technologies

Whole metabolomic methods are of growing interest in bacteriocin and RiPPs research (for an extensive recent review, see ref. 124). Peptidogenomics, which is mass spectrometry-guided genome mining for peptides, have successfully identified a novel lanthipeptide and production gene cluster from the cell extract and genome of a single strain^125^. MetaMiner has been developed to search for novel compounds from billions of mass spectra and compare with diverse metagenomic data sets such as human microbiome samples^126^. ‘Omic-mining’ can successfully identify novel peptides and biosynthetic gene clusters in an untargeted manner, but without ‘wet-laboratory’ demonstration of compound-specific antimicrobial activity, all such compounds should not be classed as bacteriocins. As the number of known biosynthetic gene clusters continues to grow, it is inevitable that biological characterization will lag behind and will have to be upscaled to explore applications of novel peptides.

## Translation from food to clinic

### Food preservatives

Fermentation as a means of food preservation, and bacteriocins unknowingly as by-products of thereof, has been utilized by humanity for millennia. Much of early bacteriocin research was performed in the context of food-fermenting organisms, such as the description of nisin in the context of delayed dairy fermentation owing to producer inhibition, and continues to be studied to this day^2,127^. Vast amounts of historical and ongoing research continue to describe the efficacy of bacteriocin producers (as fermentation starters or adjuncts) and their products to inhibit spoilage organisms in different products. However, despite decades of bacteriocin use in the food industry (nisin was designated as generally recognized as safe by the FDA in 1988 (ref. 128)) and the array of bacteriocins that have been identified to date, only a small number are currently commercially available as food preservatives (mainly nisin A, nisin Z and pediocin PA-1). Most bacteriocin-preserving agents are available as partially purified fermentates, some of which also contain the producing organism. Nisin is available as a powder with degrees of purity under the product names Nisaplin^®^ (Danisco, Dupont), Chrisin^®^ (Chr. Hansen) and vegetal or white NisinA/Z^®^ and NisinA/Z^®^ P (Handary). Pediocin PA-1 is used as an antilisterial additive in food production in a number of powdered fermentate forms such as ALTA 2351/2341 (Kerry Group) and Fargo 23^®^ (formerly in production by now defunct Quest International) approved by EFSA’s Qualified Presumption of Safety status^129^. A formulation containing *Carnobacterium maltaromaticum* CB1 and derived bacteriocins labelled Micocin^®^ (Griffith Foods) has been approved for use in North America and Canada for the preservation of meats^130^.

### Live strains and microbiome modulators

#### Microbiome modulation

Microbiome research continues to grow exponentially with considerable efforts directed towards associating particular microorganisms or microbial compositions with human diseases or conditions^131–133^. Identifying such correlations will naturally lead to attempts to develop tools for modulating the microbiome to enhance specific health-associated taxa or to reduce disease-associated taxa. Nutrition and lifestyle are obvious microbiome modulating strategies but these are essentially ‘blunt tools’, generating largely nonspecific and potentially widespread changes to gut microbiota composition and functionality. Antibiotics are also blunt tools, and despite being necessary for treatment and prevention of infectious agents, their lack of precision causes collateral damage to beneficial gut microorganisms, risking adverse health events such as *C. difficile* over-growth or the expansion of antibiotic-resistant strains^22,134,135^. This effect is not restricted to the gut, with antibiotic treatment also restructuring microbiomes of the skin, vagina and oral cavity^136–138^. Bacteriocins may prove to be an exciting alternative as potent antimicrobials, which may have narrow target spectra to target specific genera and species, and can therefore be utilized to more precisely inhibit pathogens or pathobionts, leaving benign commensals unharmed. Several class II bacteriocins have been found not to alter the overall gut microbiota composition but to reduce specific taxa such as Enterococcaceae and *Clostridium* spp. when consumed by mice^139^. Thuricin CD, produced by *B. thuringiensis*, has also been shown to specifically reduce clostridia in a faecal fermentation model, whereas vancomycin, a commonly used treatment in clinical settings, has a broad impact on the bacterial community^140^. Even broad-spectrum bacteriocins can induce only subtle changes in microbiome compositions when compared with traditional antibiotics, as was demonstrated with microcin J25 relative to rifampicin in a continuous culture porcine model^141^. Targeted microbiome modulation should also consider potential ecosystem effects as removal of a targeted species or genus could lead to unintended increases or decreases in others^142^. Simplified model ecosystems such as the OMM12 model have shown that bacteriocin production can have broad effects on neighbouring strains, and removal of a single species, in this case, a bacteriocin-producing strain, leads to the increased abundance of numerous others^143^.

#### Delivery to the gut

Therapeutics for human or animal consumption must be carefully formulated to be delivered in an active form to the correct anatomical site for function. For example, a small-molecule antibiotic must survive harsh environments during gastrointestinal transit to be absorbed and function systemically to prevent or treat infection. Bacteriocins have long been thought to be proteolytically degraded during gastrointestinal transit^144^ and therefore must be protected by encapsulation for applications directed at the large intestine. Recent evidence has challenged this dogma and shows that some bacteriocins, such as nisin, may be more resistant than previously thought, with detectable intact nisin surviving in vivo gastrointestinal transit in pigs and microcin J25 resisting degradation during simulated human digestion^37,38^. Despite this, many bacteriocins can be readily degraded by proteases in gastric transit^145,146^, and peptide delivery to the gut faces challenges of producing sufficient quantities to administer to large animals and the degree of purity required for in vivo studies. Fortunately, bacteriocins can also be deployed in the form of a probiotic or live-biotherapeutic agent that arrives at the desired destination and delivers a bacteriocin dose in situ. Researchers recently showed that genetically modified *E. coli* Nissle 1917 expressing microcin l47 in a preclinical mouse model significantly reduced carbapenem-resistant *Klebsiella pneumoniae* after 1 week oral administration of 10^8^ cells per day without alteration of the resident microbiota^34^. *E. coli* expressing the unmodified enterocins A/B and hiracin JM79 have also been shown to reduce vancomycin-resistant *Enterococcus* when provided in drinking water ad libitum (5 × 10^8^ cfu ml^−1^) in a vancomycin-resistant *Enterococcus*-colonized mouse model^14^. Live bacteriocin producers can also be used to treat or modulate microbiomes outside the gut, such as the use of an *L. lactis* strain producing lacticin 3147 in a liquid paraffin-based emulsion that was used to treat bovine mastitis (10^9^ cfu 5 ml^−1^ dose) as successfully as the standard antibiotic treatment^147^.

Bacteriocin producers that are closely related to the intended target species can have the added benefit of potentially being able to occupy the same niche, potentially providing future colonization resistance against pathogens. Bacteriocin-21-producing *E. faecalis* were found to evict their drug-resistant relatives in the mouse intestine when provided in drinking water ad libitum (5 × 10^8^ cfu ml^−1^) without perturbation of the overall community^50^. However, the plasmid-encoded nature of this bacteriocin resulted in conjugation to native enterococci providing immunity and the capacity to produce the bacteriocin. These data facilitate a scenario in which a multidrug-resistant strain could gain another tool to aid colonization and therefore must be cautiously considered when developing bacteriocin producers as targeted live biotherapeutics.

Other methods for ensuring unscathed bacteriocin delivery to site of action include through formulation with other compounds that increase peptide stability under biologically relevant conditions^148^. Silica mesoporous matrices have been utilized as protective delivery vehicles for bactofencin A and nisin A, resulting in active peptides that are resistant to trypsin^149^ and pepsin^150^ degradation, respectively. Hydrogels have been developed to ensure sustained release of bacteriocins over time, including an injectable polysaccharide gel containing nisin A active for up to 10 days^151^, and a two-phase release gel of subtilosin developed for inhibition of *Gardnerella vaginalis*, which causes bacterial vaginosis^152^. Formulation of bacteriocins into nanoparticles to increase the antimicrobial activity or widen the spectrum of the resulting nanoconjugate is a growing trend for food preservation and clinical application (reviewed elsewhere^153^). Recently, a mix of five bacteriocins adsorbed on alginate nanoparticles was found to be 500-fold more active than the peptides against clinically relevant *E. coli*^154^, and microcin J25-chitosan nanoparticles were active against enterotoxigenic *E. coli* in addition to ameliorating lipopolysaccharide-induced cytotoxicity of macrophages and downregulating lipopolysaccharide-stimulated pro-inflammatory cytokines^155^. Emerging technologies such as these should continue to improve and develop bacteriocin candidates for application in food preservation and in clinical settings.

### Clinical application

In addition to few commercial bacteriocin products on the market, none is currently approved for human therapeutic use. Research and development for clinical application of bacteriocins has been chronically underfunded, with 70 novel antimicrobials under development in 2020, of which none were bacteriocins^156^. Despite this lack of considerable investment, many preclinical and some clinical studies have been performed demonstrating that bacteriocins, and particularly lantibiotics, exert antimicrobial activity in vivo in tolerable quantities (Table 2). Several bacteriocins and their producers have been investigated for the oral treatment of *C. difficile* infection and associated diarrhoea. Preclinical trials have assessed OG-716 (mutacin derivative) for the treatment of *C. difficile* infection and associated diarrhoea when administered via oral gavage (20 mg kg^−1^ bodyweight) in golden hamsters, finding the peptide to be safe and effective at preventing relapse^107,157^. A bacteriocin-producing *Bacillus velezensis* strain as a live biotherapeutic for the treatment of *C. difficile* infection has been proven effective preclinically in mice (single oral dose of 5 × 10^8^ cfu) and in miniature swine (single oral dose of 5 × 10^8^ cfu and 28 days administration of 5 × 10^9^ cfu in capsules) and recently concluded a phase I clinical trial^158^. The semi-synthetic thiopeptide LFF571 (derived from GE2270A) has undergone phase I and II clinical trials (oral administration of 200 mg four times a day for 10 days) for *C. difficile* infections and was determined to be comparable to vancomycin treatment^159,160^.

Beyond *C. difficile* infection, the lantibiotic NAI-107 (microbisporicin) has undergone a series of preclinical rodent studies for severe antimicrobial-resistant infections with promising results^161^. In animal studies, nisin and lacticin-3147 have shown promise in the treatment of mastitis^147^, and lantibiotic-producing *Staphylococcus hominis* ShA9 decreased *S. aureus*, improving atopic dermatitis when delivered to the arms in a lotion (10^6^ cfu cm^−2^ applied twice daily for 1 week) and meeting primary and secondary endpoints in a phase I clinical trial^162^. Mutacin 1140-derived topical treatments (5 or 10 mg kg^−1^ per day for 7 days) have been proven safe and more effective than vancomycin for methicillin-resistant *S. aureus* infection in mice and have also been effective when administered intravenously (10 mg kg^−1^ per day for 6 days)^163^. Fewer trials have been performed with class II bacteriocins, but recently the effect of an Abp-118-producing *L. salivarius* live biotherapeutic on recurrent acute otitis media (middle ear infection) in children was examined^164^. The treatment was proven highly effective (84% reduction in episodes over 6 months of daily consumption of 10^9^ cfu) and as one of the most common infections in children, a commercial bacteriocin therapy could greatly reduce antibiotic prescription (Table 2).

### Beyond antimicrobials

Thiostrepton, derived from *Streptomyces* spp., has long been used in veterinary medicine for the topical treatment of acute otitis media, but has never been approved for human use nor have any other thiopeptides^165^. However, it is one of several bacteriocins gaining interest beyond antimicrobial application. Thiostrepton selectively and potently represses the cancer-associated transcriptional activator and therapeutic target, FoxM1 (ref. 166). Diverse bacteriocins have been studied in vitro and in vivo for chemotherapeutic treatment of different cancer types including nisin^167^, microcin E492 (ref. 55) and enterocin LNS18 (ref. 168), with none having yet progressed to phase I clinical trials. Moli1901 (duramycin/lancuvotide) has undergone phase I and II clinical trials as a therapeutic for cystic fibrosis as a host chloride channel activator, although with limited efficacy^169,170^. Bacteriocins are not immune to the pitfalls of drug development and can be abandoned in early clinical trials owing to the poor performance or difficulties in production. Ultimately, the pharmacokinetic and pharmacodynamic attributes of bacteriocins under investigation are critical limiting factors in enabling them to become therapeutic agents. Vastly increased funding of discovery, characterization, preclinical and human trials is required to adequately advance suitable bacteriocin candidates to the frontline of clinics, which currently continues to be sorely lacking.

## Conclusions

Bacteriocins are a diverse group of antimicrobial peptides produced across a number of bacterial phyla, and ongoing discovery efforts continue to reveal biological insights. These discoveries include not only novel peptide families but also instances of structurally similar peptides and biosynthetic machinery present in taxonomically unrelated species. This phenomenon may shed light on aspects of horizontal gene transfer and the evolution of antimicrobial peptides, akin to the convergent evolution observed in Eukaryotic defensins^171^.

Bacteriocin discovery has been transformed in recent years from the labour-intensive process of identifying producer isolates in vitro to the in silico genome mining that can yield hundreds of related peptide sequences within seconds. Machine learning has become an invaluable tool in detecting bacteriocin sequences within vast genomic data sets, and with continuous advancements in computing power and artificial intelligence training, we can expect the identification of even more novel peptides and perhaps even novel bacteriocin classes in the future. Despite the growing number of known peptides, our understanding of their mechanisms of action lags behind. Investigating the cellular targets of bacteriocins is crucial for choosing the correct antimicrobial for specific pathogenic species and for understanding resistance mechanisms. This will also enable the rational design of bacteriocins through peptide bioengineering, potentially leading to the development of highly targeted and potent therapeutic agents.

Bacteriocins hold great promise in complex microbial communities such as the gut microbiome with their ability to selectively target specific pathogens or pathobionts without disrupting the overall community structure and function. However, the potential unintended consequences of such interventions must be carefully considered to avoid disruptions to microbial ecosystems that could lead to undesirable health effects. One substantial challenge is translating laboratory discoveries into clinical applications. Bacteriocins offer a viable alternative or adjunct therapy in the treatment of infections, especially against multidrug-resistant pathogens. Realizing these opportunities will require substantial funding and rigorous clinical studies. In addition to their antimicrobial properties, structurally related peptides have been identified that lack antimicrobial activity but possess other valuable bioactivities, such as pain relief or anti-inflammatory effects^172^. These findings expand the horizon of bacteriocins beyond antimicrobials, conceivably offering multipurpose therapeutic agents.

In conclusion, bacteriocins have the potential to act as precise and versatile tools in an ongoing battle against antimicrobial-resistant microorganisms. As they continue to be explored, a path is paved whereby these remarkable molecules could have a pivotal role in safe-guarding human health and well-being. Although challenges lie ahead, the potential for bacteriocins as tools to eliminate clinically relevant microorganisms and precisely shape microbial communities is an exciting and promising avenue for research.

## Figures and Tables

**Table 1:** Suggested updated bacteriocin classification scheme with examples

Bacteriocin class^a^	Subgroup	Defining features and conserved enzyme (if known or present)	Examples
I	Bottromycin	Macrolactamidine, YcaO	Bottromycin A2 (ref. 173)
I	Cyanobactin	N-terminal proteolysis enzyme, PatA	Kawaguchipeptin B^174^
I	Darobactin	Unusual double ring structure from tryptophan-to-tryptophan, lysine or arginine linkages, rSAM	Darobactin^16^
I	Epipeptide	d-amino acids, rSAM	EpeX^175^
I	Glycocin	S-glycosylation and O-glycosylation of serine/threonine	Pallidocin^121^
I	Lanthipeptide, type I	Methyllanthionine and/or lanthionine residues, LanBC	Kunkecin A^176^
I	Lanthipeptide, type II	Methyllanthionine and/or lanthionine residues, LanM	Roseocin^177^
I	Lanthipeptide, type V (lanthidin)	Methyllanthionine and/or lanthionine residues, LanKY	Cacaoidin^178^
I	Lasso peptide	Macrolactam with threaded C-terminal tail	Ubonodin^179^
I	Linaridin	Dehydrobutyrine, no lanthionine	Corynaridin^27^
I	Linear azole-containing or azoline-containing peptide (LAP)	Azol(ine)s, YcaO	Spongiicolazolicin A/B^180^
I	Pantocin	Glu–Glu crosslink, PaaA	Pantocin A^181^
I	Pyritide (including thiopeptides)	Six-membered nitrogenous heterocycle	Thiomuracin^182^
I	Sactipeptide	Intramolecular sulfur-to-α-carbon thioether (sactionine) crosslink	Ruminococcin C^183^
I	Circular	Covalently linked N-terminal and C-terminal residues resulting in circular peptide backbone	Pumilarin^25^
I	Microcins with non-ribosomal siderophore	Serine-rich C terminus with a non-ribosomal siderophore-type modification	MccH47^184^
II	Pediocin-like	Contains YGNGVXC motif	Maltaricin CPN^31^
II	Linear, two-component	Two peptides, both required for activity	Plantaricin EF^29^
II	Linear, non-pediocin, non-two component	Lacks defining features of other groups	Bactofencin^185^
II	Defensin-like	Conserved disulfide pattern of eukaryotic defensins	Actifensin^32^
II	Leaderless	Leaderless peptides with or without four-helix motif	BacSp222, Enterocin DD14 (refs. 30,186)

rSAM, radical *S*-adenosylmethionine.

a Bacteriocins can be generally classified into two major groups: post-translationally modified (class I) and non-significantly post-translationally modified peptides (class II). Both groups are further subdivided on the basis of conserved features unique to the subgroup. Within class I bacteriocins, groups can be defined according to a specific modification that is installed by one or more modification enzymes.

**Table 2:** Bacteriocins and producers undergoing investigation for therapeutic application

Form	Bacteriocin	Class	Producer	Clinical trial phase (completed)	Treatment and animal model (if applicable)	Administration	Result	Company
Peptide	NVB333 (ref. 187)	Lantibiotic	*Actinoplanes liguriae* NCIMB41362	Preclinical	Mouse thigh infection model, MRSA disseminated	Intravenous	Equal efficacy to vancomycin	Novacta
Peptide	NVB302 (ref. 188)	Lantibiotic	*A. liguriae* NCIMB41362	Phase I	*Clostridioides difficile* infection	Oral	Not reported^189^	Novacta
Peptide	Mutacin 1140 and analogues^163^	Lantibiotic	*Streptococcus mutans* JH1000	Preclinical	Mouse MRSA skin and systemic infection	Intravenous or topical	Effective intravenous treatment	Sano Chemicals
Peptide	OG716 (mutacin 1140 derivative)^107^	Lantibiotic	*S. mutans*	Preclinical	Hamster model of *Clostridioides difficile* infection and associated diarrhoea	Oral	100% survival and no relapse	–
Peptide	NAI-107 (microbisporicin)^190^	Lantibiotic	*Microbispora corallina*	Preclinical	Acute severe infections in mouse and rat models	Intravenous	Lower effective dose than best alternative, rapid bactericidal activity	Naicons SRL, Sentinella Pharmaceuticals
Peptide	Moli1901 (duramycin/lancuvotide)^169,170^	Lantibiotic	*Streptomyces cinnamoneum*	Phases I and II	Cystic fibrosis symptoms	Intranasal, inhalation	Well tolerated, no difference from placebo	Lantibio/AOP Orphan Pharmaceuticals
LBT	Sh-lantibiotic-α/β (ref. 162)	Lantibiotic	*Staphylococcus hominis* ShA9	Phase I	Atopic dermatitis	Topical	Achieved primary safety end point and decreased *Staphylococcus aureus*	–
LBT	Abp-118 (ref. 164)	Pediocin-like	*Ligilactobacillus salivarius* PS7	Phase I	Recurrent acute otitis media	Oral consumption	Significant decrease (84%) in AOM episodes	ProbiSearch
LBT	Multiple^158^	Multiple	*Bacillus velezensis* ADS024	Preclinical	*C. difficile* infection	Oral	Inhibited *C. difficile* infection in mice and did not colonize mice or swine after 28-day repeat dose	Adiso Therapeutics

AOM, acute otitis media; LBT, live biotherapeutic; MRSA, methicillin-resistant *Staphylococcus aureus*.
